# *Klf5* suppresses ERK signaling in mouse pluripotent stem cells

**DOI:** 10.1371/journal.pone.0207321

**Published:** 2018-11-19

**Authors:** Takuya Azami, Ken Matsumoto, Hyojung Jeon, Tsuyoshi Waku, Masafumi Muratani, Hitoshi Niwa, Satoru Takahashi, Masatsugu Ema

**Affiliations:** 1 Department of Stem Cells and Human Disease Models, Research Center for Animal Life Science, Shiga University of Medical Science, Seta, Tsukinowa-cho, Otsu, Shiga, Japan; 2 Department of Anatomy and Embryology, Faculty of Medicine, University of Tsukuba, Tsukuba, Ibaraki, Japan; 3 Graduate School of Pharmaceutical Sciences, The University of Tokyo, Hongo, Bunkyo-ku, Tokyo, Japan; 4 Department of Genome Biology, Faculty of Medicine, University of Tsukuba, Tsukuba, Ibaraki, Japan; 5 Department of Pluripotent Stem Cell Biology, Institute of Molecular Embryology and Genetics, Kumamoto University, Chuo-ku, Kumamoto, Japan; 6 International Institute for Integrative Sleep Medicine (IIIS), University of Tsukuba, Tsukuba, Ibaraki, Japan; 7 Life Science Center (TARA), University of Tsukuba, Tsukuba, Ibaraki, Japan; 8 Transborder Medical Research Center (TMRC), University of Tsukuba, Tsukuba, Ibaraki, Japan; 9 Laboratory Animal Resource Center (LARC), University of Tsukuba, Tsukuba, Ibaraki, Japan; 10 PRESTO, Japan Science and Technology Agency, Saitama, Japan; JAPAN

## Abstract

Mouse embryonic stem cells (ESCs) are pluripotent stem cells, which have the ability to differentiate into all three germ layers: mesoderm, endoderm, and ectoderm. Proper levels of phosphorylated extracellular signal-regulated kinase (pERK) are critical for maintaining pluripotency, as elevated pERK evoked by fibroblast growth factor (FGF) receptor activation results in differentiation of ESCs, while, conversely, reduction of pERK by a MEK inhibitor maintains a pluripotent ground state. However, mechanisms underlying proper control of pERK levels in mouse ESCs are not fully understood. Here, we find that Klf5, a Krüppel-like transcription factor family member, is a component of pERK regulation in mouse ESCs. We show that ERK signaling is overactivated in *Klf5*-KO ESCs and the overactivated ERK in *Klf5*-KO ESCs is suppressed by the introduction of *Klf5*, but not *Klf2* or *Klf4*, indicating a unique role for *Klf5* in ERK suppression. Moreover, *Klf5* regulates *Spred1*, a negative regulator of the FGF-ERK pathway. *Klf5* also facilitates reprogramming of EpiSCs into a naïve state in combination with a glycogen synthase kinase 3 inhibitor and LIF, and in place of a MEK inhibitor. Taken together, these results show for the first time that Klf5 has a unique role suppressing ERK activity in mouse ESCs.

## Introduction

Pluripotent stem cells (PSCs) can be established as embryonic stem cells (ESCs) in culture from the epiblast of a blastocyst [1,2]. PSCs can also be generated as induced pluripotent stem cells (iPSCs) through the induction of pluripotency from somatic cells by ectopic expression of defined factors such as Oct3/4, Sox2, Klf4, and c-Myc [3]. Pluripotency of mouse ESCs is regulated by extracellular stimuli such as leukemia inhibitory factor (LIF) [4], as well as nuclear factors such as Oct3/4, Sox2, and Nanog [5–9]. Pluripotency is also achieved by the combinatorial inhibition of extracellular signal-regulated kinase (ERK) signaling and glycogen synthase kinase 3β (GSK3β), called the ground state [10]. Conversely, extracellular stimuli elicited by fibroblast growth factor (FGF) activates the ERK pathway in mouse ESCs, thereby destabilizing the pluripotent state and promoting cellular differentiation [10–12].

Mouse epiblast stem cells (EpiSCs) are PSCs derived from post-implantation epiblast at E5.5, the egg cylinder stage [13,14]. Although EpiSCs retain the ability to differentiate into all three germ layers, EpiSCs hardly contribute to fetal tissues when injected into a blastocyst. Mouse EpiSCs and human ESCs share many properties such as gene expression patterns, epigenetic modifications, proliferative activities, and cytokine responsiveness [15]. Both EpiSCs and human ESCs depend on basic FGF and activin signaling for self-renewal, indicating that responsiveness of the FGF-ERK pathway is substantially different between mouse ESCs and human ESCs. PSCs capable of contributing to a chimera are defined to be in a naïve state, while PSCs that depend on FGF signaling and are incapable of contributing to chimeras are in a primed state [15]. A substantial number of studies have demonstrated that naïve PSCs differentiate into primed PSCs [16,17], while primed PSCs can be converted into the naïve state by defined factors such as Nanog and Klf2 [18–20].

Krüppel-like transcription factor family members (Klfs) such as Klf2, Klf4, and Klf5 have important functions in both maintenance of mouse ESC pluripotency and the cellular reprogramming process [16,21–26]. Previous studies clearly demonstrated an association between expression of Klfs and naïve pluripotency, and the self-renewal capacity of mouse ESCs was severely reduced when *Klf2*, *Klf4*, and *Klf5* were knocked down [21] or knocked out (KO) [26]. While *Klf2*, *Klf4*, and *Klf5* have redundant functions in the maintenance of pluripotency, our previous report indicated that *Klf5*-KO mouse ESCs exhibit a spontaneous differentiation phenotype [22]. Our recent study demonstrated overactivation of the ERK pathway in *Klf5*-KO preimplantation embryos and promotion of primitive endoderm specification of inner cell mass cells at the expense of epiblast in *Klf5*-KO embryos [27]. However, it is unknown whether *Klf5* regulates FGF-ERK pathway in mouse ESCs.

Here, we show overactivation of ERK in *Klf5*-KO ESCs. Importantly, such overactivation is suppressed by the introduction of *Klf5*, but not *Klf2* or *Klf4*, indicating a unique role of *Klf5* in ERK suppression. *Klf5* regulates *Spred1*, a negative regulator of the FGF-ERK pathway. *Klf5* facilitates reprogramming of EpiSCs into a naïve state in combination with a GSK3 inhibitor and LIF, and in place of MEK inhibition. Taken together, our results demonstrate for the first time that Klf5 has a unique role in suppressing ERK activity in mouse ESCs.

## Results

### Loss of *Klf5* results in an increased level of pERK in mouse ESCs

**Fig 1:**
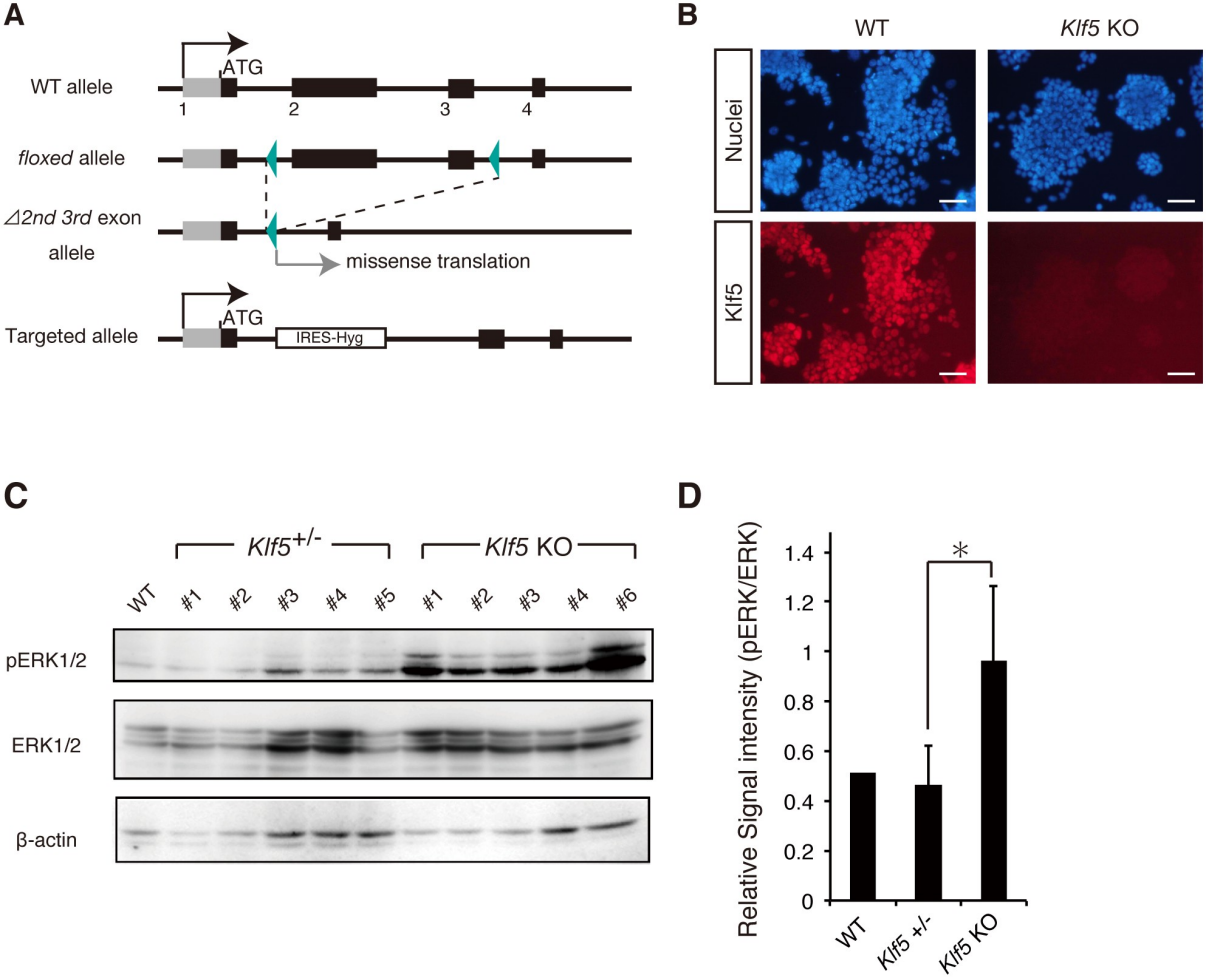
Klf5 suppresses pERK in mouse ESCs. (A) Schematic representation of *Klf5* targeting vector to create *Klf5*-KO ESCs. (B) Expression of Klf5 protein in WT and *Klf5*-KO ESCs. Scale bar: 100 μm. (C) Western blot analysis of WT, *Klf5* heterozygous, and *Klf5*-KO ESCs. Western blot analysis was performed with antibodies against pERK, ERK, and β-actin. (D) Quantified signal intensity for pERK relative to ERK. Asterisk indicates statistical significance: *P < 0.01; Mann-Whitney U test.

Proper levels of pERK are critical for maintaining pluripotency, yet how pERK levels are properly controlled in mouse ESCs is not fully understood. As our recent study showed that the ERK pathway is repressed by the transcription factor Klf5 in preimplantation mouse embryos [27], we examined whether *Klf5* regulates ERK signaling in mouse ESCs. To evaluate pERK levels in only Oct3/4-positive PSCs, new *Klf5*-KO ESC lines were generated from Oct3/4-CFP::Rex1-GFP (OCRG9) ESCs in which a puromycin resistance gene was introduced into the *Oct3/4* locus (Fig 1A and 1B), thereby enabling rapid selection of Oct3/4-positive PSCs [28]. After culture in the presence of puromycin, we performed western blotting analysis and found that pERK levels were significantly elevated in *Klf5*-KO ESC lines compared with WT and *Klf5* heterozygous ESCs (Fig 1C and 1D).

### *Klf5*, but not *Klf2* or *Klf4*, rescues elevated pERK levels in *Klf5*-KO ESCs

**Fig 2:**
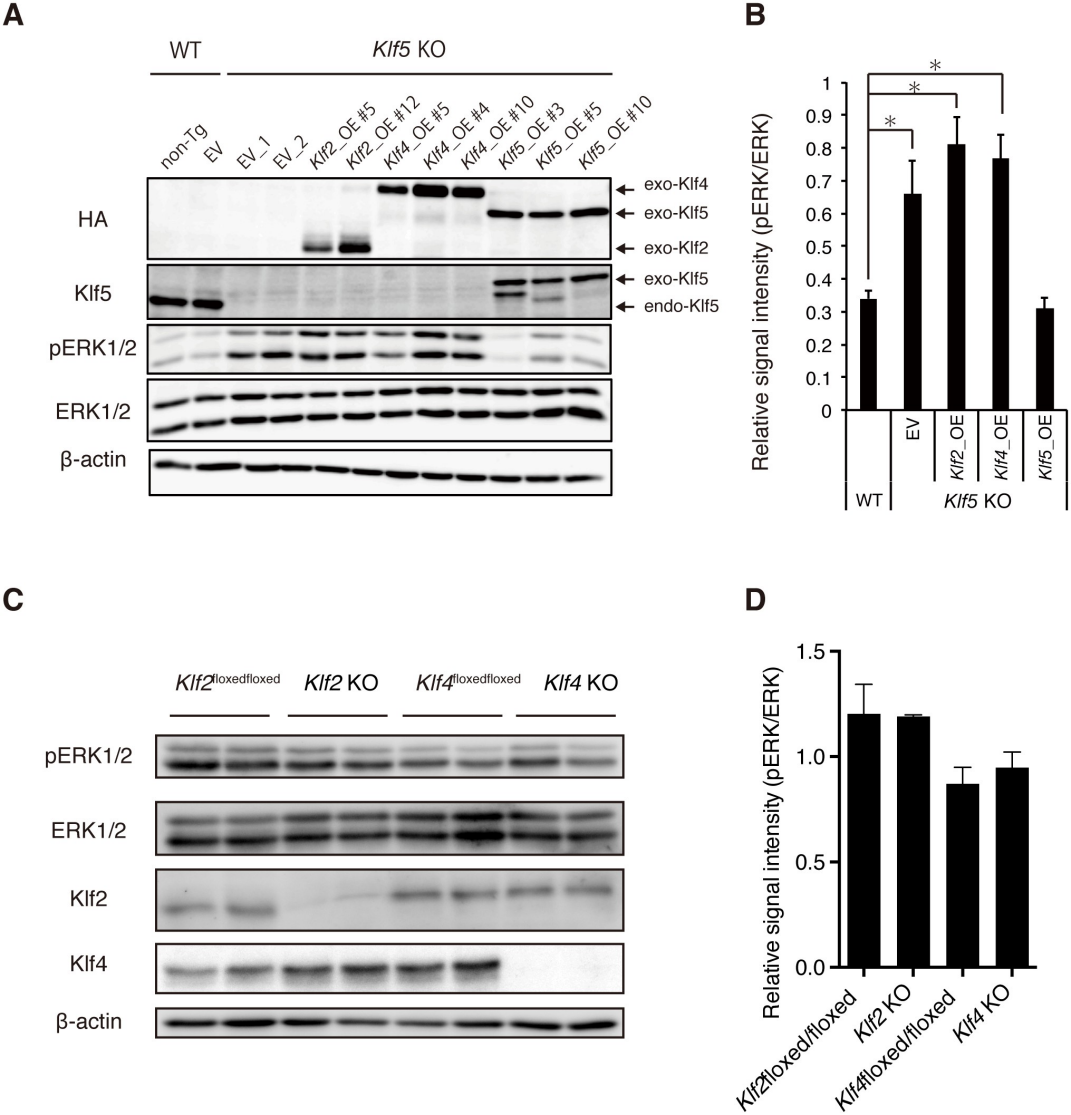
Rescue of elevated pERK levels in *Klf5*-KO ESCs by *Klf5*, but not *Klf2* or *Klf4*. (A) Level of pERK in *Klf5*-KO ESCs overexpressing *Klf2*, *Klf4*, or *Klf5*. Western blot analysis of WT non-transgenic (non-Tg), empty vector (EV) control, *Klf5*-KO EV control, Klf2-overexpressing (Klf2_OE), Klf4_OE, and Klf5_OE ESCs were performed with antibodies against HA, Klf5, pERK, ERK, and β-actin. Exo; exogenous, endo; endogenous. (B) Quantified signal intensity for pERK relative to ERK. (C) Level of pERK in *Klf2*-or *Klf4*-KO ESCs. Western blot analysis of WT, *Klf2*-KO ESCs, and *Klf4*-KO ESCs. (D) Quantified signal intensity of pERK relative to ERK. Asterisk indicates statistical significance: *P < 0.01; Mann-Whitney U test.

Previous reports showed that *Klf* family members such as *Klf2*, *Klf4*, and *Klf5* have redundant functions to maintain the pluripotency of mouse ESCs [21,22,26]. To investigate whether *Klf2*, *Klf4*, and *Klf5* have similar functions on pERK regulation in mouse ESCs, epitope-tagged versions of Klf2, Klf4, or Klf5 were introduced into *Klf5*-KO ESCs and respective Klf-Tg ESC lines were established (Fig 2A). Expression levels of epitope-tagged Klf were similar to endogenous Klf5 expression in WT ESCs (Fig 2A). Elevated pERK levels in *Klf5*-KO ESCs were reversed to normal levels in WT ESCs by the introduction of Klf5, but not Klf2 or Klf4 (Fig 2A and 2B), indicating a unique Klf5 function on suppression of pERK. To more directly investigate whether Klf2 and Klf4 are involved in pERK regulation, we examined pERK levels in *Klf2-* or *Klf4*-KO ESCs by western blot analysis; no significant changes were observed (Fig 2C and 2D). Collectively, these results demonstrate a unique role for *Klf5* in pERK suppression in mouse ESCs.

**Fig 3:**
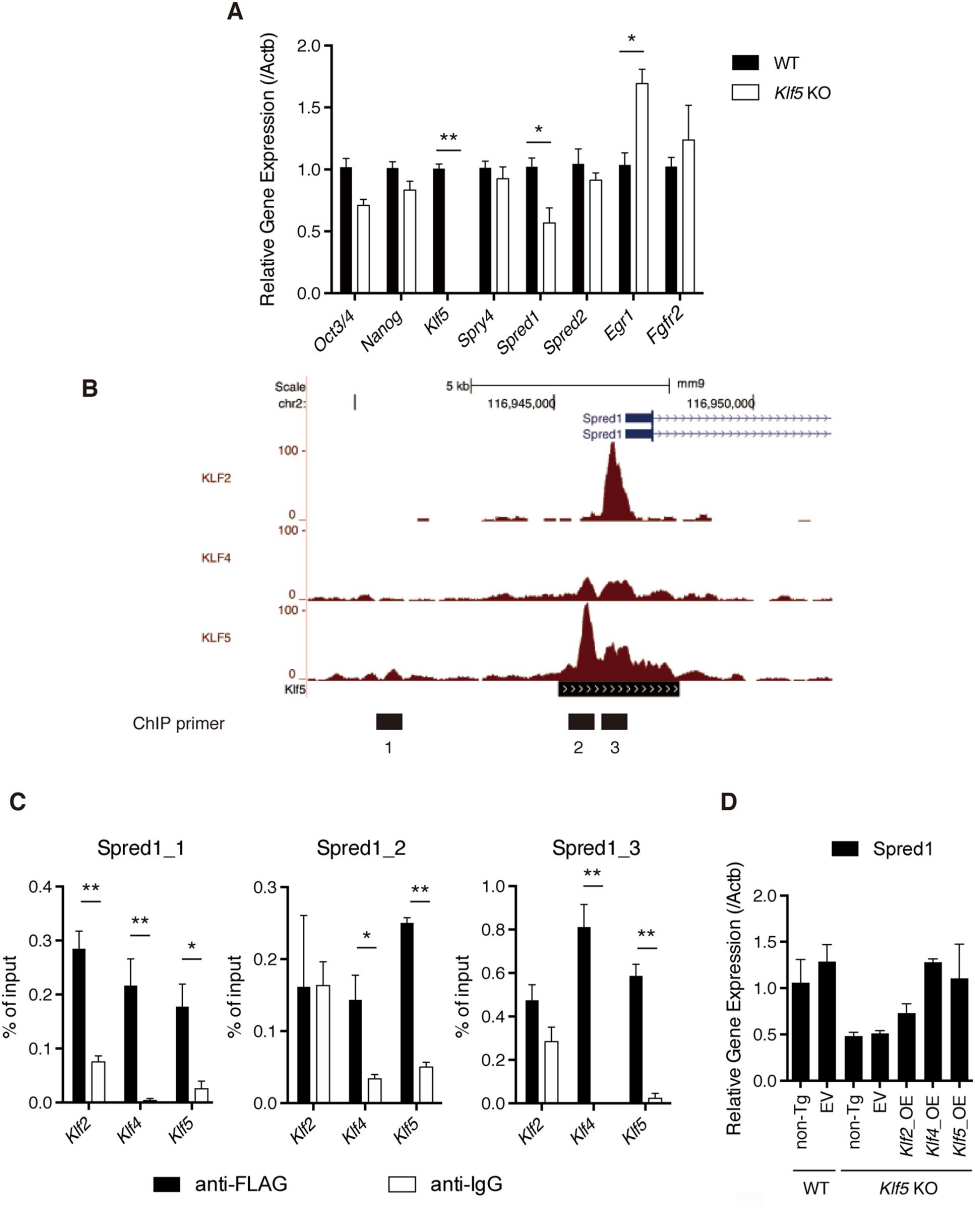
*Klf5* regulates *Spred1* in mouse ESCs. (A) RT-qPCR analysis of genes involved in the FGF-FGFR-ERK pathway. (B) Binding peaks of Klf2, Klf4, and Klf5 to *Spred1* locus. Numbers below binding peaks indicate regions for ChIP primers. (C) Manual ChIP assay of Klf2, Klf4, and Klf5 binding to *Spred1* in mouse ESCs. (D) *Spred1* mRNA expression in *Klf5*-KO ESCs overexpressing *Klf2*, *Klf4*, or *Klf5*. Asterisks indicate statistical significance: *P < 0.01, *P* < 0.001** (Mann-Whitney U test).

Given that pERK was elevated in *Klf5*-KO ESCs, we assessed whether expression of genes involved in the FGF-FGFR-ERK pathway were altered in *Klf5*-KO ESCs. RT-qPCR analysis indicated that *Egr1* is induced in *Klf5*-KO ESCs, consistent with the notion that *Egr1* is a direct target of ERK [29] and *Spred1* [30], a suppressor for ERK that was also significantly reduced in *Klf5*-KO ESCs (Fig 3A). To investigate whether *Klf5* binds to *Spred1* directly, we surveyed genomic binding sites of *Klf5* by examining ChIP-seq data and found that *Spred1* was occupied by Klf5 (Fig 3B). To confirm this finding, we used ESC lines overexpressing epitope-tagged Klf5, and found that Klf5 bound preferentially to *Spred1* loci (Fig 3C). These results indicated that Klf5 regulates *Spred1* to maintain proper pERK levels in mouse ESCs. Notably, *Spred1* was also occupied by Klf2 and Klf4 (Fig 3B and 3C), and the introduction of *Klf2* or *Klf4* significantly rescued *Spred1* expression (Fig 3D). These results indicated that Klf2 and Klf4 also have ability to activate transcription of *Spred1*. However, given that the elevated pERK level in *Klf5*-KO ESCs was reversed by Klf5, but not Klf2 or Klf4, we speculate that there may be other FGF-FGFR-ERK pathway-related molecules regulated by Klf5, but not Klf2 or Klf4.

**Fig 4:**
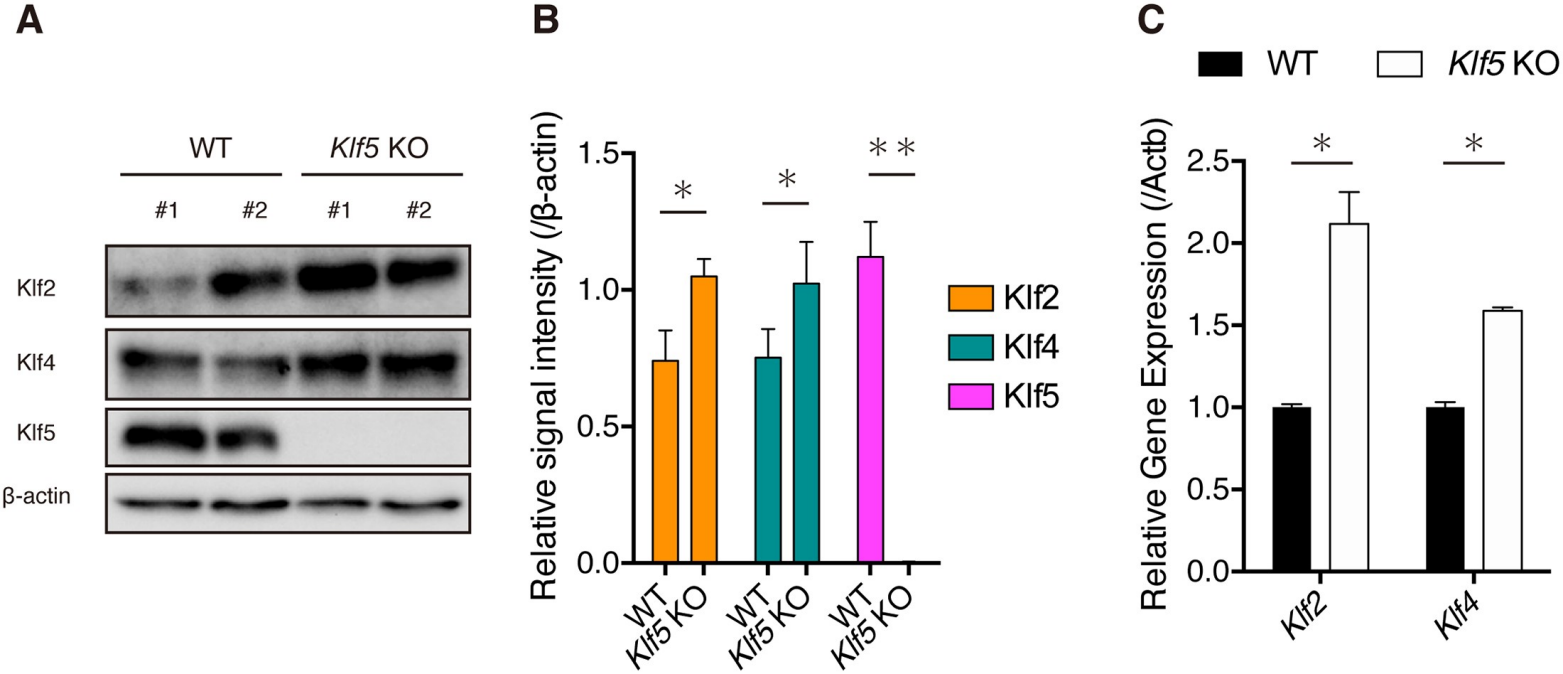
Levels of Klf2 and Klf4 in *Klf5*-KO ESCs. (A) Western blot analysis of WT and *Klf5*-KO ESCs. Western blot analysis was performed with antibodies against Klf2, Klf4, and β-actin. (B) Quantified signal intensity of Klf2 or Klf4 relative to β-actin. (C) RT-qPCR analysis of *Klf2* and *Klf4* in WT and *Klf5*-KO ESCs.

Previous studies demonstrated that both Klf2 and Klf4 proteins are phosphorylated by ERK1/2 before proteolysis by the proteasome-degradation pathway [25,31]. As pERK levels were elevated in *Klf5*-KO ESCs, we examined whether levels of Klf2 and Klf4 proteins were reduced (Fig 4A). Unexpectedly, levels of both Klf2 and Klf4 protein were significantly increased in *Klf5*-KO ESCs (Fig 4A and 4B). Thus, we examined mRNA levels and found that both *Klf2* and *Klf4* mRNA were significantly increased in *Klf5*-KO ESCs (Fig 4C). We speculate that compensatory upregulation of *Klf2* and *Klf4* mRNA may lead to an increase in both proteins, although degradation of *Klf2* and *Klf4* proteins may be promoted by elevated pERK.

### Klf5 facilitates reprogramming in combination with GSK3β inhibition

**Fig 5:**
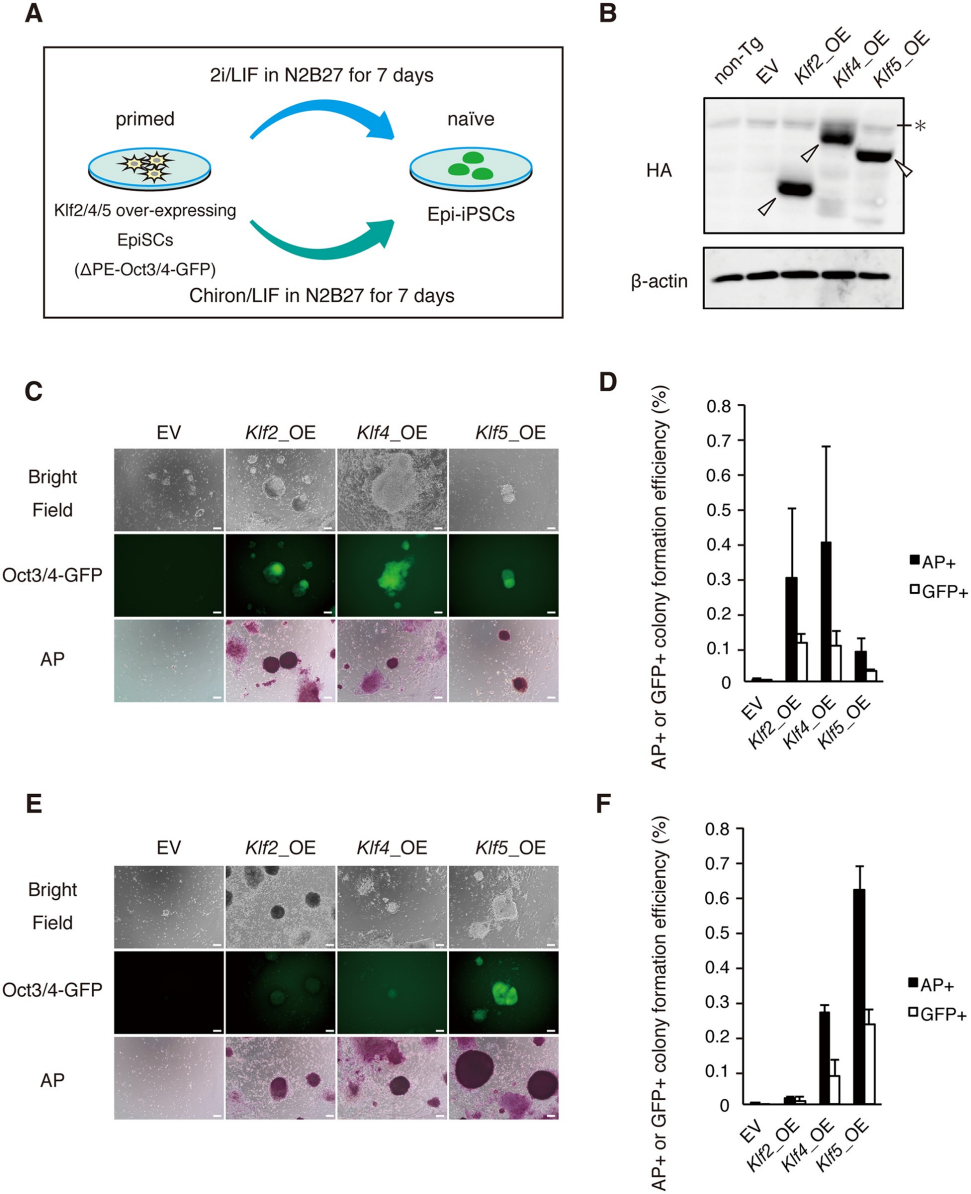
*Klf5* facilitates reprogramming towards naïve pluripotency in combination with Chiron. (A) Experimental design to evaluate the reprogramming activity of Klf proteins. EpiSCs (3 × 10^4^) were replated onto fibronectin-coated dishes and cultured in N2B27 medium containing 2i/LIF or Chiron/LIF. After 7 days in 2i/LIF or Chiron/LIF, iPSC colonies were picked for an AP assay. (B) Expression levels of epitope-tagged Klf2, Klf4, and Klf5 in EpiSCs. Open arrowheads indicate epitope-tagged Klf proteins. * indicates non-specific band. (C) Reprogramming ability of Klf2, Klf4, and Klf5 in the presence of 2i and LIF. Scale bar: 100 μm. (D) Comparison of reprogramming efficiency among *Klf2*, *Klf4*, and *Klf5* in the presence of 2i by AP assay and counting of GFP-positive colonies. (E) Reprogramming ability of Klf2, Klf4, and Klf5 in the presence of Chiron and LIF. Scale bar: 100 μm. (F) Comparison of reprogramming efficiency among *Klf2*, *Klf4*, and *Klf5* in the presence of Chiron by AP assay and counting of GFP-positive colonies.

Mouse EpiSCs can be reprogrammed into Epi-iPSCs by induced expression of naïve transcription factors, such as Nanog and Esrrb, in the presence of 2i [16,20,32]. This system is widely used to evaluate the reprogramming ability of a putative reprogramming factor (Fig 5A). Previous studies reported that Klf2 and Klf4 have strong reprogramming activity from fibroblasts into iPSCs, whereas Klf5 has rather weak activity [16,20,24]. Similarly, *Klf2* and *Klf4* efficiently (0.1%–0.2%) facilitated the reprogramming of EpiSCs towards Epi-iPSCs, in contrast to *Klf5* (0%) [20]. We reassessed the effect of Klf2, Klf4, and Klf5 on reprogramming using EpiSCs overexpressing epitope-tagged versions of these Klf proteins, and confirmed that all three proteins were expressed at similar levels (Fig 5B). First, we examined the efficiency of reprogramming in a typical reprogramming medium [N2B27 medium containing a MEK inhibitor and GSK3ß inhibitor (2i)] by alkaline phosphatase (AP) assay and green fluorescent protein (GFP) fluorescence driven by ΔPE-Oct3/4 transgene activity, which is only present in naïve PSCs. It was observed that Klf2 and Klf4 had strong reprogramming activities (0.3%–0.5%) (Fig 5C and 5D). *Klf5* also had moderate activity (0.1%) (Fig 5C and 5D), consistent with our previous work [24]. Given that *Klf5* represses the FGF-ERK pathway in pre-implantation embryos [27] and suppresses pERK in mouse ESCs (current study), we presumed that *Klf5* might facilitate the reprogramming process in place of a MEK inhibitor. Thus, we cultured EpiSCs in the absence of a MEK inhibitor, and found that Klf5 generated a substantial number of Oct3/4-positive colonies with greater efficiency than Klf2 or Klf4 (Fig 5E and 5F).

Notably, the reprogramming efficiency elicited by Klf5 without MEK inhibition was higher than with MEK inhibition (Fig 5D and 5F). Recently, Yagi et al. and Choi et al. reported that prolonged culture in the presence of 1 μM MEK inhibitor causes loss of DNA methylation in mouse pluripotent stem cells, which erases of genomic imprinting and alters developmental potential into embryonic lineages [33,34]. Therefore, we speculated that treatment with 1 μM MEK inhibitor in combination with Klf5 may cause an additive effect for downstream genes, thereby reducing the reprogramming efficiency; although, the exact mechanism is currently unclear.

**Fig 6:**
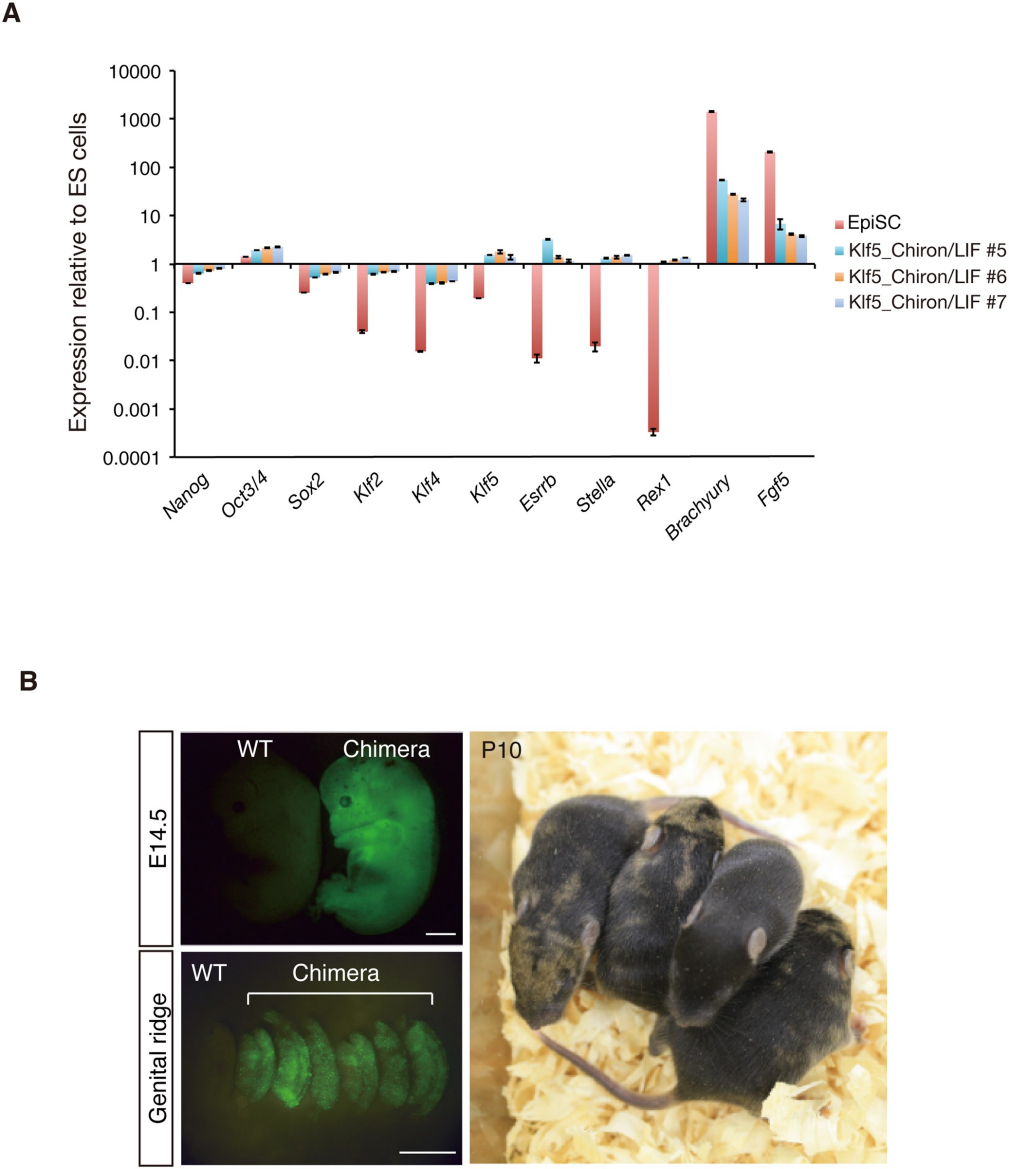
Chimera-forming ability of EpiSCs reprogrammed by Klf5 with Chiron/LIF. Reprogrammed Epi-iPSCs show the hallmarks of naïve pluripotency. (A) RT-qPCR analysis of expression of markers for naive pluripotency and differentiation. After the Klf5 expression cassette was removed by transient Cre expression, RT-qPCR analysis was performed. Levels of expression relative to those in ESCs are shown. (B) Chimera generated from Epi-iPSCs induced by Klf5 in combination with Chiron. Upper panels show a control WT embryo and embryo injected with GFP-labeled Epi-iPSCs on the left and right, respectively. Lower panel shows a postnatal chimera at P10 injected with GFP-labeled Epi-iPSCs. Note that brown coat color indicates the contribution of 129 Epi-iPSCs. Plotted results are mean and SEM of three independent experiments. Scale bar: 100 μm.

As Oct3/4-positive colonies exhibited very similar gene expression patterns to ESCs (Fig 6A), we performed blastocyst injections to examine the contribution of Oct3/4-positive cells to post-implantation embryos (Fig 6A and 6B). Injected embryos showed strong contributions to embryonic and postnatal chimeras (Fig 6B). These results suggest that Klf5 facilitates reprogramming in collaboration with GSK3β in the absence of a MEK inhibitor by regulating the FGF-ERK pathway.

## Discussion

Although proper pERK levels are known to be required for maintenance of pluripotency, how pERK levels are properly controlled in mouse ESCs is not well understood. Thus far, extrinsic BMP4 attenuates ERK activity by inducing the ERK-specific phosphatase DUSP9 to promote self-renewal [35,36]. The MYC/MAX complex also suppresses ERK activity by regulating Dusp2 and Dusp7 to maintain the naïve state of PSCs, as a lack of MAX causes defective self-renewal and differentiation accompanied by ERK activation [37,38]. Our recent study showed that the transcription factor *Klf5* represses the ERK pathway via suppression of *Fgf4* in preimplantation mouse embryos [27]. Although we previously showed that *Fgf4* is not altered in *Klf5*-KO ESCs [27], current our study clearly indicates that Klf5 suppresses pERK in mouse ESCs via *Spred1*, a negative regulator for ERK signaling.

Previous reports have indicated that *Klf2*, *Klf4*, and *Klf5* have redundant functions in the self-renewal of mouse ESCs and in the induction of pluripotency [23]. However, it appears that *Klf4* and *Klf5* have differential roles on cellular proliferation [22]. Yeo et al. reported that Klf2 is directly phosphorylated by Erk2, and phospho-Klf2 undergoes proteasome-dependent degradation [25]. Therefore, inhibition of MEK can prevent Klf2 phosphorylation and stabilize Klf2 protein, thereby activating genes related to naïve pluripotency, which explains in part how MEK inhibition promotes the formation of naïve PSCs. Similarly, ERK phosphorylates Klf4 as part of the proteasome pathway [31]. In this regard, it is interesting to note that pERK was elevated in *Klf5*-KO ESCs and increased pERK could be reversed by overexpression of *Klf5*, but not *Klf2* or *Klf4*. Thus, these data clearly demonstrate a unique role for *Klf5* in the suppression of pERK in ESCs.

Taken together, our results clearly demonstrate that Klf5 is a critical genetic component that suppresses MEK activity in naïve PSCs.

## Materials and methods

### Pluripotent stem cells

Mouse *Klf5* hyg/floxed ESC lines were created by introducing a *Klf5* hygromycin-resistant gene knock-in vector [22] and floxed *Klf5* vector [27] into OCRG9 ESCs (Oct3/4-CFP::Rex1-GFP-irespuroR, a generous gift from Dr. Niwa). Subsequently, pCAG-NLS-Cre (a generous gift from Dr. Andras Nagy, Samuel Lunenfeld Research Institute) was transiently introduced to delete the second and third exons of *Klf5*, thus generating *Klf5*-KO (hyg/Δ) ESC lines. ESCs were cultured in Dulbecco’s Modified Eagle’s Medium (DMEM) containing 10% fetal bovine serum (FBS) in the presence of puromycin to enrich Oct3/4-positive PSCs. *Klf2*-floxed ESCs and *Klf4*-floxed ESCs were described previously [39].

OZ7 mouse EpiSCs, a generous gift from A. Smith [16], were maintained in N2B27 medium (Stem Cells Sciences) supplemented with 12 ng/μL basic fibroblast growth factor (bFGF) and 20 ng/μL activin A (R&D Systems). To assay the reprogramming of EpiSCs, an effector plasmid expressing *Klf2*, *Klf4*, or *Klf5* was transfected into OZ7 cells using Lipofectamine 2000 (Invitrogen) and cells were cultured on fibronectin-coated dishes. Subsequently, EpiSCs (3 × 10^4^) were replated onto fibronectin-coated dishes and cultured in N2B27 medium containing 2i (1 μM PD0325901 and 3 μM CHIR99021)/LIF, or 3 μM Chiron/LIF. After 7 days in 2i/LIF or Chiron/LIF, iPSC colonies were picked or an AP assay was performed using an AP staining kit (Sigma-Aldrich).

### Immunohistochemistry and AP assay

PSCs were fixed in 4% paraformaldehyde for 10 min, permeabilized in 0.5% Triton X-100 for 10 min, and incubated in a blocking reagent [phosphate-buffered saline (PBS) containing 0.1% bovine serum albumin and 0.01% Tween 20] for 1 hr. PSCs were incubated at 4°C overnight with primary antibodies (S1 Table). After three washes with PBS containing 0.2% Tween 20, secondary antibodies were incubated at room temperature for 1 hr. Nuclei were stained with Hoechst 33342 (10 μg/mL, Molecular Probes). AP assays to identify PSCs were performed with a leukocyte AP staining kit (Sigma-Aldrich).

### Blastocyst injection

The Klf5 expression unit was removed from Epi-iPSC lines reprogrammed from 129 EpiSCs, which were cultured in DMEM + 10% FBS and transfected with pPB-UbC-GFP plasmid and a transposase, as described in Jeon et al. [24]. GFP-positive Epi-iPSCs were harvested and injected into C57BL/6 mouse blastocysts (SLC Inc., Shizuoka, Japan), and chimeras were dissected out at E9.5 and E13.5. Some chimeras were analyzed for their coat color at postnatal day 10. The sacrifice was carried out by cervical dislocation. The experimental procedures were approved by the ethics committee for Animal Experimentation of Shiga University of Medical Sciences (Approval number: 2016-11-8) (Committee members are Akira Andoh, Jun Udagawa, Masatsugu Ema, Kazumasa Ogasawara, Shinichiro Nakamura, Kazuhiko Nozaki, Seiji Hitoshi, Kihachiro Horiike, Yoshihito Muroji, Shigehiro Morikawa). Masatsugu Ema was not involved in the judgement.

### Quantitative PCR analysis

For RT-PCR analysis, first-strand cDNA was synthesized from total RNA using a QuantiTect Reverse Transcription kit (Qiagen). Real-time PCR was performed with SYBR Premix Ex Taq II (TaKaRa) and analyzed on a Thermal Cycler Dice Real Time System (TP850; TaKaRa). Amounts of target RNA were estimated using an appropriate standard curve and normalized by dividing values by the estimated amount of β-actin. Sequences of primers used for quantitative PCR are listed in S2 Table.

### Western blot analysis

ESCs were lysed and western blotting was performed as described previously [22]. Membranes were immunoblotted with rat anti-mouse Klf5 antibody (KM1784; Kyowa Kirin), rabbit anti-pERK antibody (Cell Signaling Technology), rabbit anti-ERK antibody (Cell Signaling Technology), rabbit anti-Klf2 antibody (Millipore), rabbit anti-Klf4 antibody (Abcam), or anti-β-actin antibody (MBL), followed by an appropriate secondary antibody [horseradish peroxidase-conjugated rabbit anti-mouse IgG (Invitrogen) or horseradish peroxidase-conjugated goat anti-rabbit IgG (Zymax)]. Immunoreactive proteins were detected using enhanced chemiluminescence (Chemilumi One; Nakalai) and an ImageQuant LAS 4000 imager (GE Healthcare). Signal intensity of western blotting was quantified using ImageJ. Signals were normalized to the intensity of ERK or β-actin.

### Chromatin immunoprecipitation (ChIP)-sequencing data analysis

Data analysis was performed as described previously [27]. ChIP-seq data were downloaded from the EMBLE-EBI site [Klf2: ERR440998, Klf4: SRR952210, Klf5: SRR952211, Aksoy et al., 2014]. ChIP-seq reads were located to the mouse reference genome (mm9) using Burrows-Wheeler alignment software. Uniquely mapped reads were used for peak calling by CCAT3 version 3.0. Peak regions were filtered for false discovery rate values < 0.05. RefSeq genes that had a Klf5 binding site within 20 kb were identified by determining the overlap between ChIP-seq peak regions and RefSeq genes extended by 20 kb in both directions. To visualize ChIP-seq tag counts in the UCSC Genome Browser, mapped reads were extended and converted into the bedGrapgh format using the genomecov function of BEDTools.

### Chromatin immunoprecipitation assays

ChIP assay was performed as described previously [27]. Sequences of primers used for ChIP-qPCR are listed in S3 Table.

### Statistical analysis

Statistical analyses were performed using the Mann-Whitney U test. Data are expressed as mean and standard error (SEM). Differences were considered significant at *P* < 0.05.

## Supporting information

S1 Fig Raw data for western blot analysis. Uncropped western blot images shown in Figures are presented.
(PDF)

S2 Fig Raw data for western blot analysis. Uncropped western blot images shown in Figures are presented.
(PDF)

S1 Table List of antibodies used in this study. Antibodies used in this study are presented.
(PDF)

S2 Table List of qRT-PCR primers. Primer sequences for qRT-PCR analysis are presented.
(PDF)

S3 Table List of ChIP-qPCR primers. Primer sequences for ChIP-qPCR analysis are presented.
(PDF)
