# Supplementary figures and images for: Klf5 suppresses ERK signaling in mouse pluripotent stem cells
Source: PLoS One. 2018 Nov 19;13(11):e0207321. doi: 10.1371/journal.pone.0207321 (PMC6242311; doi:10.1371/journal.pone.0207321)

## S1 Fig

Uncropped western blot images for all figures as indicated.

Fig. 1C

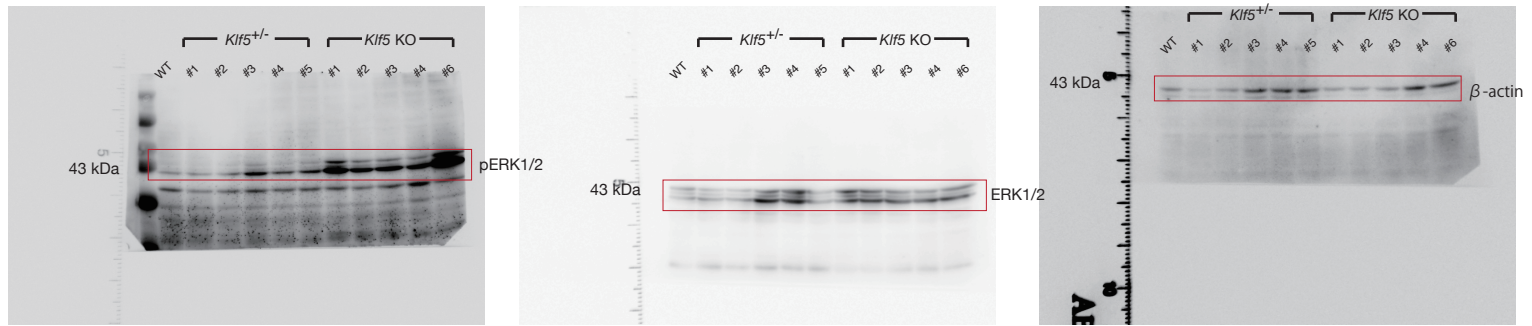

Fig. 2A

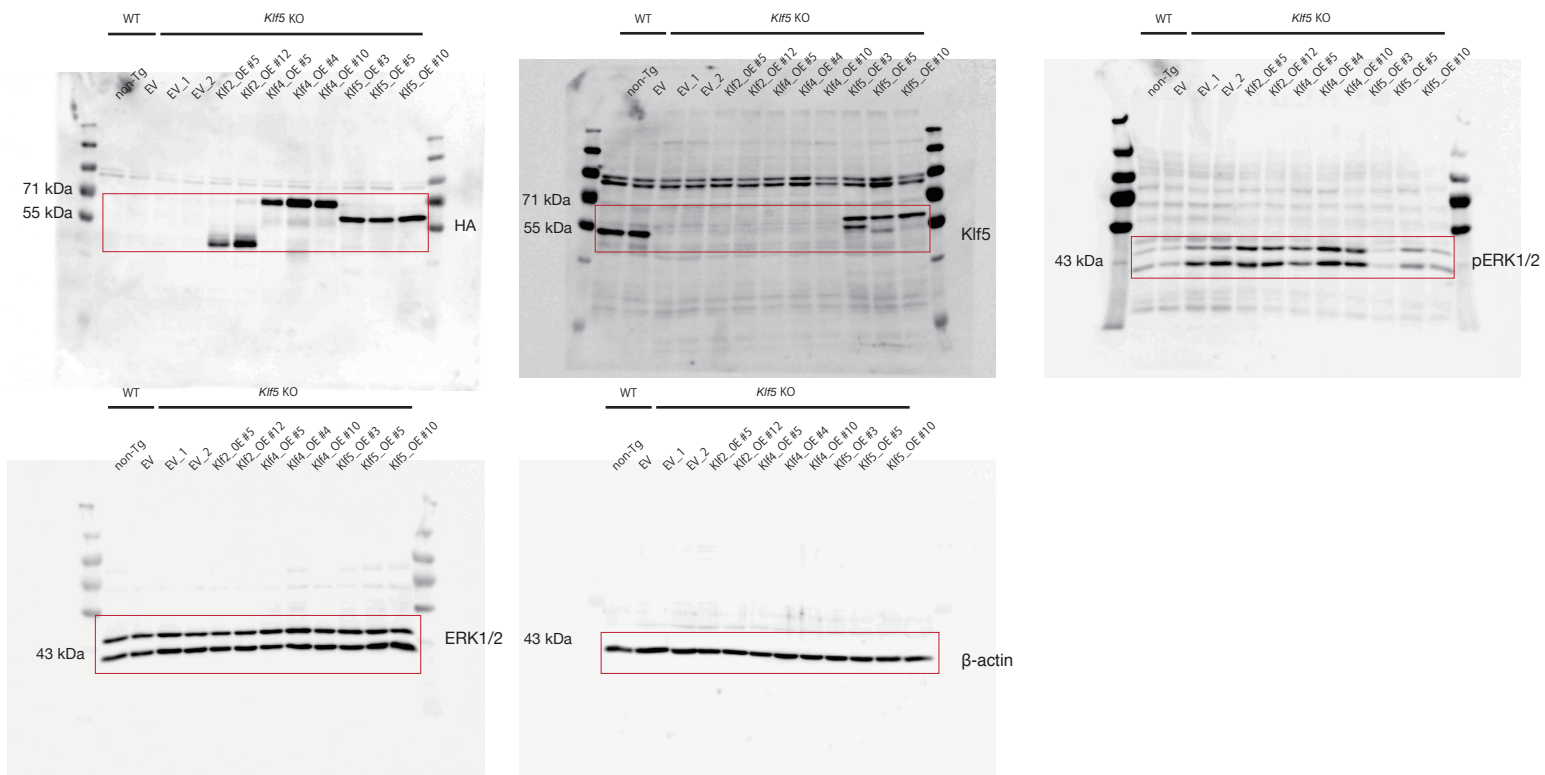

Fig. 2C

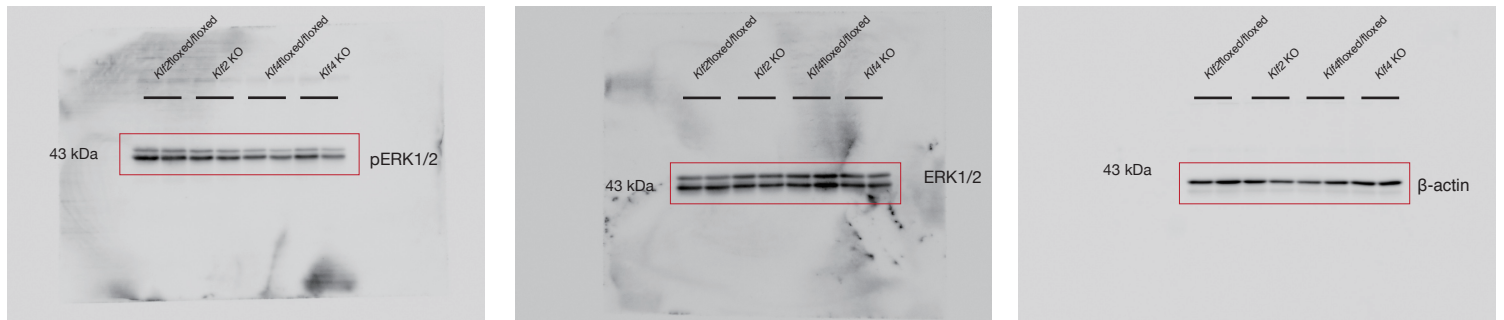

Supplement: S1 Fig — Uncropped western blot images shown in Figures are presented. (PDF) [file pone.0207321.s001.pdf]

## S2 Fig

Uncropped western blot images for all figures as indicated.

Fig. 2C

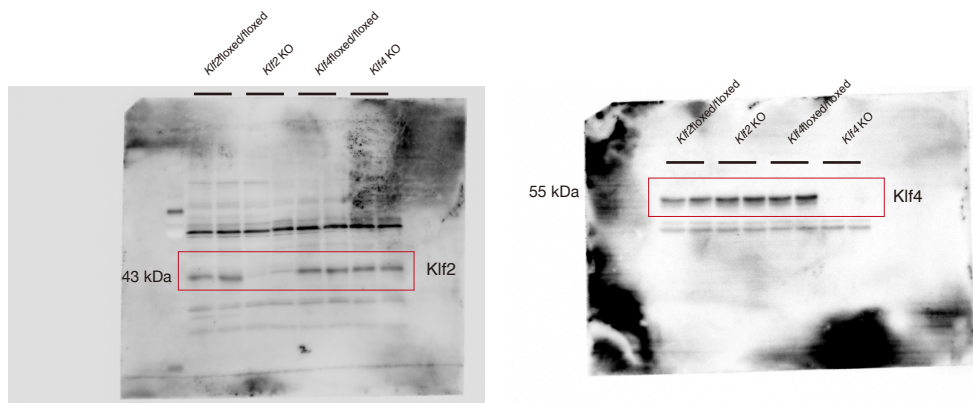

Fig. 4A

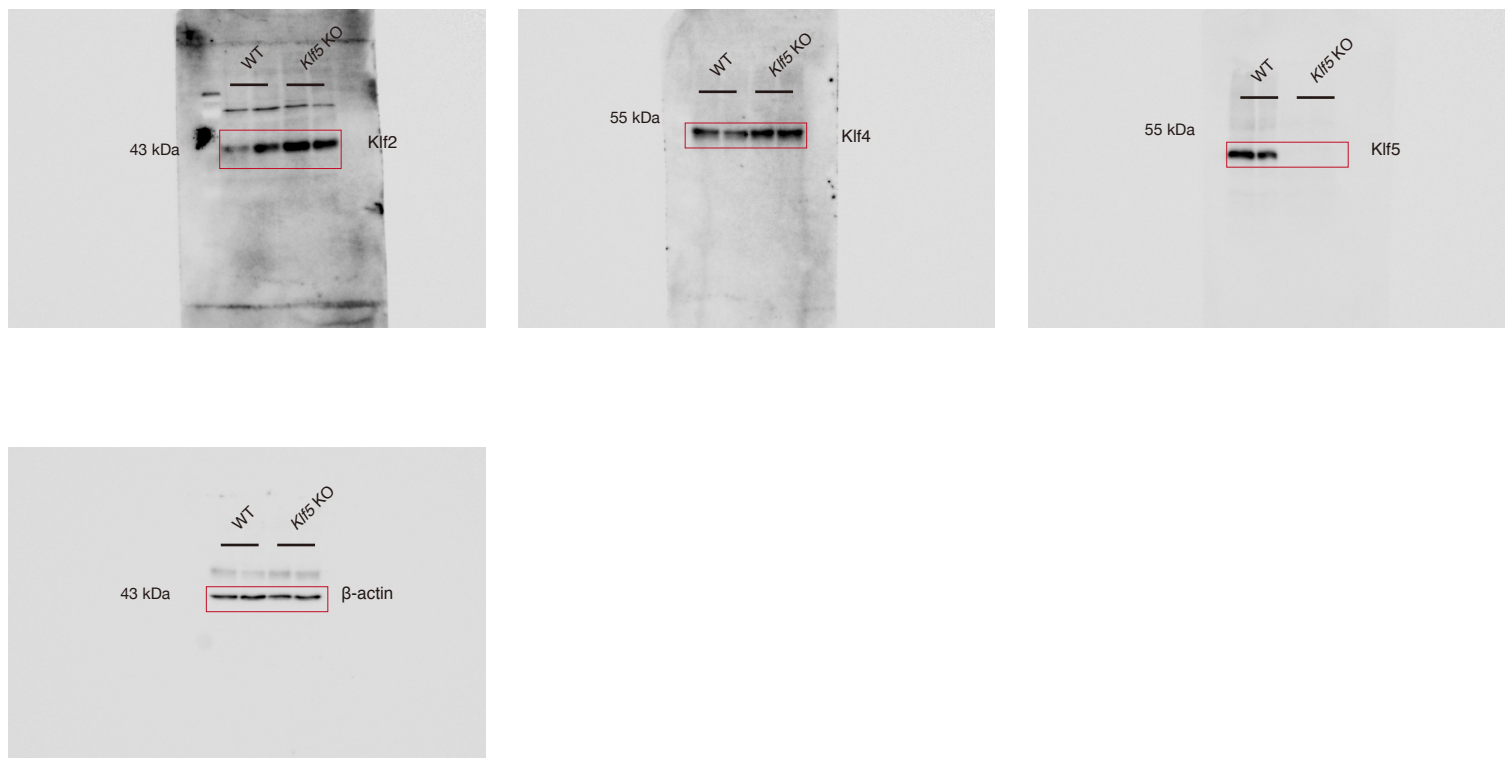

Fig. 5B

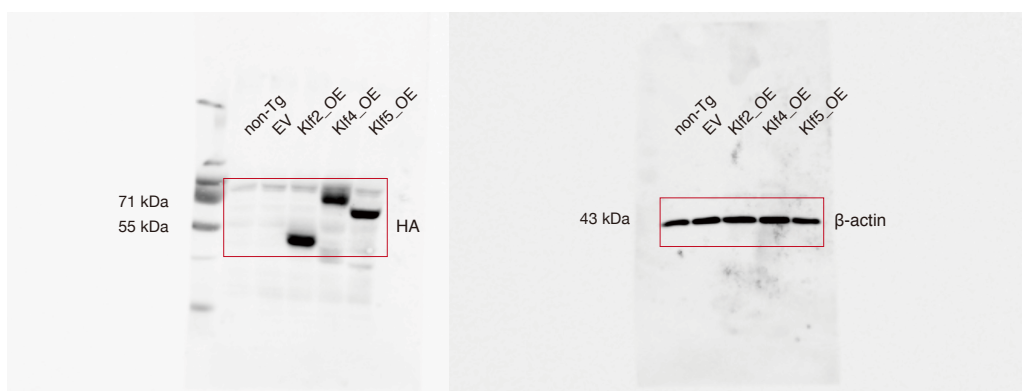

Supplement: S2 Fig — Uncropped western blot images shown in Figures are presented. (PDF) [file pone.0207321.s002.pdf]
