# Supplementary material for: Klf5 suppresses ERK signaling in mouse pluripotent stem cells
Source: PLoS One. 2018 Nov 19;13(11):e0207321. doi: 10.1371/journal.pone.0207321 (PMC6242311; doi:10.1371/journal.pone.0207321)
Supplement: S1 Table — Antibodies used in this study are presented. (PDF) [file pone.0207321.s003.pdf]

S1 Table: List of antibodies

Primary Antibody	Supplier	Catalogue Number	Dilution
rabbit anti-pERK1/2	Cell Signaling Technorogy	#9101	1:1000
rabbit anti-ERK1/2	Cell Signaling Technorogy	#9102	1:2000
rat anti-HA	Roche	1867423001	1:2000
anti-FLAG-M2	sigma	F1804	1:250
anti-β-actin HRP	MBL	PM053-7	1:5000
rat anti-Klf5	Kyowa KIRIN	a gift from Dr. Nagai	WB; 1:1000, IF; 1:300
rabbit anti-Klf2	Millipore	09-820	1:1000
rabbit anti-Klf4	abcam	ab215036	1:1000
