# Supplementary material for: Klf5 suppresses ERK signaling in mouse pluripotent stem cells
Source: PLoS One. 2018 Nov 19;13(11):e0207321. doi: 10.1371/journal.pone.0207321 (PMC6242311; doi:10.1371/journal.pone.0207321)
Supplement: S2 Table — Primer sequences for qRT-PCR analysis are presented. (PDF) [file pone.0207321.s004.pdf]

S2 Table: List of qRT-PCR primers sequences

Gene	Primer Sequence (Forward)	Primer Sequence (Reverse)
Spred1	TTAGCTGCATGCTCTGTGCT	ACAGCGATGGCACATTCTCA
Egr1	TTGTGGCCTGAACCCCTTTT	AGATGGGACTGCTGTCGTTG
Dusp1	GGGAGCTGGTCCTTATTTATTTAAC	CTCAAACAGAGTCCTTTCTCTTCTG
Nanog	ACAGGTTTCAGAAGCAGAAGTACC	AATCAGACCATTGCTAGTCTTCAAC
Oct3/4	TATTGAGTATTCCCAACGAGAAGAG	CTCAGGAAAAGGGACTGAGTAGAGT
Sox2	CATGAGAGCAAGTACTGGCAAG	CCAACGATATCAACCTGCATGG
Klf2	GCGTACACACACAGGTGAGA	GCACAAGTGGCACTGAAAGG
Klf4	TACCCCTACACTGAGTCCCG	GGAAAGGAGGGTAGTTGGGC
Klf5	CGATTCACAACCCAAATTTACC	GTATGAGTCCTCAGGTGAGCTTTTA
Esrrb	GGACTATATCATGGATGAGGAACAC	TTCCTTCTCTACCTTGAGTTTCTTG
Stella	AGGCTCGAAGGAAATGAGTTTG	TCCTAATTCTTCCCGATTTTCG
Rex1	TCCATGGCATAGTTCCAACAG	TAACTGATTTTCTGCCGTATGC
Brachyury	ATCAGAGTCCTTTGCTAGGTAG	GTTACAATCTTCTGGCTATGC
Fgf5	ACCCACTTCCTACCCAGGTT	AGTTGTTTCCCACAAGGCCA
β-actin	CTGAGCGCAAGTACTCTGTGTG	GTGTAAAACGCAGCTCAGTAACAGT
