# Supplementary material for: Klf5 suppresses ERK signaling in mouse pluripotent stem cells
Source: PLoS One. 2018 Nov 19;13(11):e0207321. doi: 10.1371/journal.pone.0207321 (PMC6242311; doi:10.1371/journal.pone.0207321)
Supplement: S3 Table — Primer sequences for ChIP-qPCR analysis are presented. (PDF) [file pone.0207321.s005.pdf]

S3 Table: List of ChIP-qPCR primers sequences

Gene	Primer Sequence (Forward)	Primer Sequence (Reverse)
Spred1_1	AGAAAGCTTGCCAACTGTGG	GCTCTGGAGGTTGCTACTCG
Spred1_2	AACGTGACAGCTGCCTATCC	TTACATGAATCTGGCGCATC
Spred1_3	CACTGAGCAGTACGGAGTGG	GAGGGATATGTCGCTGCACT
